# Recent advancements in penile prosthetics

**DOI:** 10.12688/f1000research.17407.1

**Published:** 2019-02-18

**Authors:** Mathew Q Fakhoury, Joshua Halpern, Nelson Bennett

**Affiliations:** 1 Department of Urology, Cook County Hospital, Chicago, IL, USA; 2 Department of Urology, Northwestern University, Chicago, IL, USA

**Keywords:** Erectile dysfunction, Penile prosthesis, Surgery

## Abstract

Since the original inflatable penile prosthesis in the 1970s, several enhancements to penile prosthesis implant design, implant surgical technique, and post-operative care have been developed to increase overall patient (and partner) satisfaction rates. We, in this communication, seek to discuss these advancements and the overall impact in combating erectile dysfunction. As we continue to pursue avenues of effective and definitive treatment modalities for erectile dysfunction refractory to medical therapy, rates of infection and mechanical failure will hopefully continue to decline in the perioperative setting.

## Introduction

Since its initial description by Scott *et al*. in 1973, the penile prosthesis has remained a mainstay of treatment of erectile dysfunction (ED) ^1^. Insertion of a penile prosthesis is an excellent option for men with ED refractory to pharmacotherapy or those men who wish to forego pharmacotherapy in favor of a permanent solution. Indeed, both patient and partner satisfaction is highest for penile prosthesis relative to alternative treatments for ED ^2– 4^. Accordingly, the American Urological Association recommends that all men with ED be informed regarding penile prosthesis as a potential treatment option ^5^.

Over the past four decades, penile prostheses have undergone numerous iterations and advancements. The arrival of the inflatable penile prosthesis (IPP) led to a cascade of device innovation over the ensuing years. Cylinder material and design have evolved to improve durability while maximizing penile length and girth ^6^. Of note, options for increases in tube length—“Optimized Tubing Length” by Boston Scientific (Marlborough, MA, USA)—have allowed greater surgical flexibility, as the input tubing increases in length with the length of the cylinder placed. (One to five centimeters of additional length of tubing is offered.)

Implants impregnated with minocycline and rifampin have helped to reduce the risk of infection following implantation ^7, 8^. Similarly, devices with hydrophilic coating allow absorption of aqueous antibiotic solutions, enabling the surgeon to select an individualized antibiotic regimen and thereby decreasing bacterial adherence ^7, 8^. Evolution of pump design, such as the one-touch release, has optimized the patient experience, rendering the devices easier to use ^9^. Two- and three-piece IPPs allow individualized device selection that incorporates patient-specific anatomic considerations. Lock-out valve reservoirs have virtually eliminated the risk of auto-inflation ^10^.

Advances in the devices themselves have been paralleled by expanded indications and improved techniques for implantation. In recent years, prosthetics have been used for not only ED but also treatment of Peyronie’s disease, penile deformity, and even penile augmentation ^11, 12^. Moreover, a number of innovative surgical techniques have emerged to improve the safety, efficacy, and cosmesis of implantation. These include the subcoronal approach, ectopic reservoir placement, concurrent scrotoplasty, and many others ^13, 14^. In many instances, the technology has evolved to accommodate these surgical innovations such as low-profile reservoirs devised for optimal submuscular ectopic placement ^15, 16^. Furthermore, adjunctive concomitant procedures have been employed with promising results. In a subset of patients with both post-prostatectomy ED, as well as climacturia or mild stress urinary incontinence (or both), placement of a “Mini-Jupette” graft provides a gentle urethral lift intended to provide improvement in continence ^17^.

In patients with both ED and Peyronie’s disease, placement of a penile prosthesis may not correct a severe penile curvature. In those cases, plaque incision with grafting (PIG) during IPP placement will be necessary. Tachosil (Baxter International, Deerfield, IL, USA) is a new grafting material that is coated with tissue sealant. It is simply pressed over the tunical defect for several seconds without the need to suture it in place ^18^.

Most recently, there have been a number of new advancements in penile prosthetics related to peri-operative pain management, surgical technique, and device innovation. This review aims to examine these specific areas of improvement in penile prosthetics.

## Peri-operative pain management

The opioid epidemic in the US has dramatically altered the approach to peri-operative pain management. Surgeons have been implicated in one of the many factors contributing to the opioid epidemic: over-prescribing post-operative narcotics for surgical pain management ^19^. Indeed, about 1 in 1,111 urological surgery patients will develop opioid dependence or overdose ^20^. As such, urologists have begun to devise multi-modal approaches to the management of post-operative pain for a variety of urological procedures ^21– 23^.

A number of studies have reported the use of novel, intra-operative local anesthetic regimens to improve post-operative pain management and reduce narcotic utilization following penile prosthesis placement. Reinstatker *et al*. performed a retrospective analysis of intra-operative dorsal penile nerve block using an extended-release bupivacaine liposomal suspension (Exparel ^®^, Pacira Pharmaceuticals, Inc., Parsippany-Troy Hills, NJ, USA), which led to substantially lower utilization of narcotic pills in the experimental versus control group (8.2 versus 24.1 tablets, *P* <0.001) ^24^. Likewise, Cotta *et al*. found that men receiving an extended-release bupivacaine liposomal suspension had significantly decreased narcotic use after implantation relative to those who did not ^25^. However, the authors reported significantly higher costs in the extended-release bupivacaine group, which may limit its routine use ^25^. A variety of other local anesthetic approaches (dorsal penile nerve, pudendal nerve, crural, and intracorporal) and medications (lidocaine, bupivacaine, and ropivacaine) have been studied and employed with varying success ^24, 26^.

Most recently, Tong *et al*. described a multi-modal analgesic (MMA) protocol that uses a series of pre-operative, intra-operative, and post-operative interventions to optimize peri-operative pain control and minimize narcotics ^27^. Pre-operatively, patients on protocol received 975 mg acetaminophen, 300 mg gabapentin, and 7.5 or 15 mg meloxicam prior to induction of anesthesia. Intra-operatively, a combination dorsal penile and pudendal nerve block was performed using a mixture of 1% lidocaine and 0.5% bupivacaine prior to incision. Post-operatively, patients were administered 975 mg acetaminophen every 6 hours, 300 mg gabapentin every 8 hours, and 7.5 or 15 mg meloxicam daily. Compared with those not on the MMA protocol, MMA patients were discharged home with fewer narcotics (mean 12.7 versus 51.3 tabletss, *P* <0.001) and required fewer narcotic refills (11% versus 49%, *P* = 0.007) ^27^. Though limited by its retrospective nature and small sample size, this study suggests a growing role for an MMA approach in penile prosthetics.

## Operative advances

Preservation of penile length is important to both the patient and the surgeon. Unfortunately, many men want to have the penile girth and length that they experienced as an adolescent. Patients with decreased penile length and girth have higher rates of dissatisfaction and decreased quality of life. Men commonly associate the length of their penis with their degree of masculinity ^28^. As a result, surgical enhancements have been introduced to preserve penile length.

Ventral phalloplasty (release of penoscrotal webbing) in combination with prosthetic implant has increased in popularity as it enhances the perceived length of the penis. Release of the penoscrotal web has been shown to enhance the patient perception of increased penile length and further improve satisfaction as reported by Miranda-Sousa *et al*. ^29^. A more recent technique, dorsal phalloplasty, has also been described in order to increase visible penis length by using permanent sutures to tack the dermis and pre-pubic fat to the pubic symphysis. Shaeer *et al*. reported a 23% increase in visible length in the dorsal phalloplasty group ^30^. Of those who underwent simultaneous phalloplasty and implant placement, only 6.1% reported penile shortening compared with 80% in the prosthesis-only group ^30^.

Ziegelmann *et al*. recently reported on a modified glanulopexy technique for correcting supersonic transporter (SST) deformity and glandular hypermobility (GH) in men undergoing IPP implantation ^31^. The authors note that this technique to correct SST/GH after IPP placement had no reported impact on penile sensation. Positively, the small incision required does not necessitate manipulation of Buck’s fascia compared with previously reported techniques for SST deformity in the setting of IPP placement.

For patients with severe penile shortening, Rolle *et al*. introduced the sliding technique in order to maintain pre-operative length in which the penis is essentially transected mid-shaft and elongated with the assistance of a penile prosthesis adding an estimated increase of up to 3.2 cm ^32^. The sliding technique was later revised into the modified sliding technique (MoST) and the multiple slide technique (MUST). However, Wilson *et al*. demonstrated a 33% rate of glans necrosis after performance of a sliding technique for penile lengthening; thus, most implanters are now avoiding the technique ^33^. Although length may be preserved, it is proposed that the necessary mobilization of the neurovascular and urethra during the sliding technique may compromise distal penile circulation ^33^.

Other adjunctive measures have been pioneered with the goal of penile length and girth preservation while preventing prosthetic surgical complications such as glans necrosis, mentioned previously. Ediygo’s multiple-slit technique was subsequently introduced as an evolution of the aforementioned techniques (MoST) ^34^. Herein, the tunica defects created during the sliding maneuver are not covered with a graft, highlighting a crucial difference previously described. More specifically, the MUST results in multiple smaller tunical defects as opposed to two large tunica defects. This development may also overcome potential bulging of cylinders by distributing the defect among multiple small slices. In those patients that have narrowed penile girth, placing of multiple longitudinal slits into the tunica albuginea will allow the penis to regain its natural girth ^34^.

## Future directions

In Plato’s *Republic*, Socrates states: “Necessity is the mother of invention”. A need or problem encourages creative efforts to meet the need or solve the problem. Both Boston Scientific and Coloplast (Humlebæk, Denmark) have continually improved components of their devices. Currently, modifications of the IPP pump are being developed to assist with inflation of the prosthesis. Additionally, researchers are developing advanced drug-eluting cylinder materials that may decrease the rate of infection. Similarly, a few companies are developing the electronically activated artificial urinary sphincter ^35, 36^. This implanted device would be activated by a mobile telephone application to facilitate voiding. Suffice it to say that the development of the electronic version of the penile prosthesis is on the horizon.

The most dreaded complication of penile prosthetic implantation is infection. Current guidelines recommend removal of the prosthesis followed by irrigation of the penis and scrotum and a lengthy the course of antibiotics. Unfortunately, if another device is not immediately placed, the patient develops corporal fibrosis and then penile shortening. Carrion recently developed a synthetic plaster-like vancomycin/tobramycin cast which is inserted into the infected corporal space to facilitate clearance of offending bacteria ^37^. This calcium sulfate internal cast has also been shown to prevent penile shortening while preserving the intracorporal space for future implantation of a prosthesis ^37^.

Investigators are beginning to think about the mechanics of penile prosthetics in new ways. Traditionally, IPPs have been based on a hydraulic phenomenon. Prostheses in development rely on the expansion and contraction of metal alloys to create a rigid erection. The implant is a nickel-titanium–based shape memory alloy that is heat-activated and alternates between a flaccid and erect configuration solely by the application of heat (that is, a heating pad) or cold (that is, an ice pack). Le *et al*. were able to demonstrate that this prosthesis can produce the mechanical forces necessary for producing a penetration-quality erection comparable to that produced by hydraulic-based devices ^38^.

## Conclusions

As a result of the numerous enhancements of the IPP over the last 40 years, as well as, improved techniques for implantation, the IPP, when compared to other types of implant, is the less likely to need surgical revision. In a comparison of prosthetics in urology with implants used in the orthopedic, cardiac, ophthalmology, and breast realms, the 10- and 15-year revision-free survival rates demonstrated that the IPP is one of the most dependable medical devices implanted into humans, as reported by Wilson *et al*. ^39^. Most recently, a number of advances have led to reduced infection rates, device durability, improved outcomes, and better patient satisfaction. To date, IPP offers the highest patient satisfaction of any ED treatment available, and the continued innovation in this field aims to ensure that this remains true for years to come.
